# A Pilot Study on the Effectiveness of Education in Supporting Diabetes Management among Patients with Severe Mental Illness

**DOI:** 10.1192/j.eurpsy.2026.10913

**Published:** 2026-07-31

**Authors:** L. N. N. Dube, L. Moore

**Affiliations:** 1 Psychiatry, University Hospital Waterford, Waterford; 2 Psychiatry, St. Luke’s General Hospital, Kilkenny, Ireland

## Abstract

**Introduction:**

Diabetes is a chronic illness that has become increasingly more prevalent in the past three decades. People with severe mental illness are estimated to be three times more likely to develop diabetes than the general population and are also at higher risk of complications. This survey was conducted amongst a cohort of patients under the care of the Recovery and Rehabilitation service in Waterford, all of whom have a diagnosis of a severe enduring mental illness and are treated with psychotropic medications. A number of the patients are diagnosed with type 2 diabetes. A need was identified within the service to improve patient knowledge and management of diabetes.

**Objectives:**

To assess the effectiveness of a structured education session in improving patients’ knowledge of managing diabetes.

**Methods:**

A pilot survey was completed to assess the effectiveness of a diabetes education intervention. The rehabilitation team had developed a diabetes handbook in collaboration with staff and patients, that gave information on diabetes and how it is managed. Patients with type 2 diabetes were identified and they completed a questionnaire to assess their baseline knowledge of diabetes and its management. They were then invited to complete an information session given over two days, based on the handbook. They were then asked to complete a second survey post session to measure any change in knowledge.

**Results:**

20 patients were identified that meet the inclusion criteria. 14 patients participated in both sessions, while 6 patients attended only one session. The mean questionnaire score at baseline for the patients who attended both sessions was 8. The score improved to 9.2 after the second session. Results are provided in Table 1.

**Image 1:**
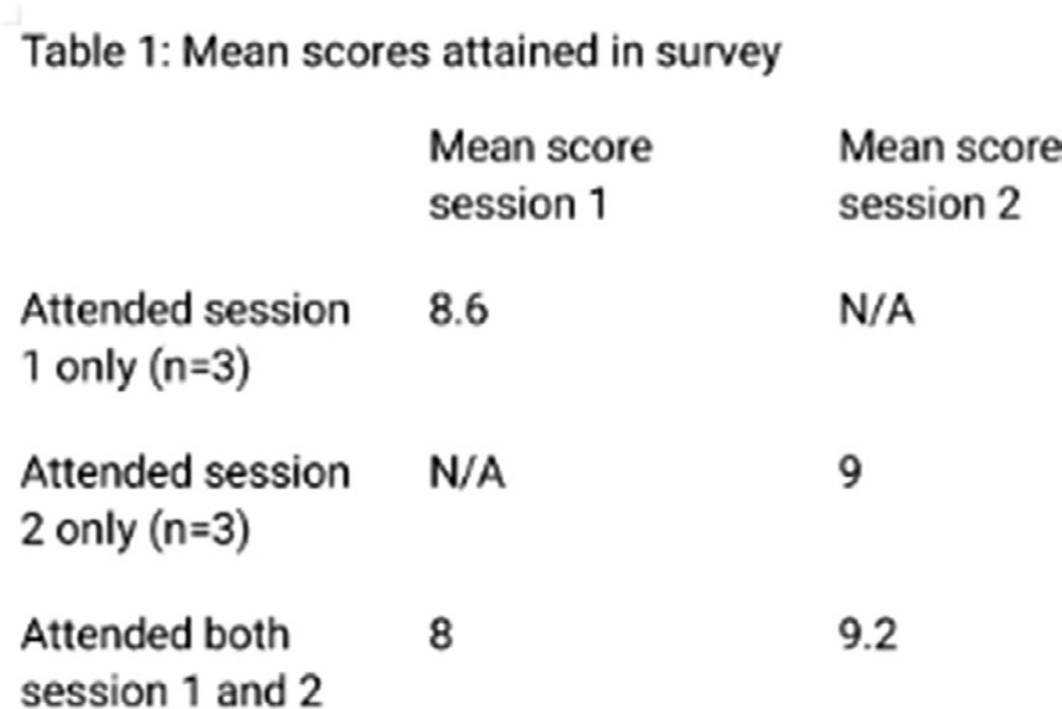

**Conclusions:**

This survey showed that a short teaching programme can be effective in improving diabetes knowledge among psychiatric patients under the rehab service. Regular diabetes education incorporated into the rehabilitation programme would likely help both support self-management and improve long term health outcomes.

**Disclosure of Interest:**

None Declared

